# Gastric paraganglioma: a case report and review of literature

**DOI:** 10.3389/fonc.2024.1357612

**Published:** 2024-04-02

**Authors:** Chengyu Hu, Bixian Luo, Bo Hong, Mingqi Zhang, Zelai Wu, Xiuliang Zhu, Fengming Luan, Yi Huang, Weihua Gong

**Affiliations:** ^1^ Department of Surgery, The Second Affiliated Hospital of Zhejiang University School of Medicine, Hangzhou, China; ^2^ Department of Pathology, The Second Affiliated Hospital of Zhejiang University School of Medicine, Hangzhou, China; ^3^ Department of Radiology, The Second Affiliated Hospital of Zhejiang University School of Medicine, Hangzhou, China

**Keywords:** gastric paraganglioma, diagnostic, immunohistochemistry, catecholamine crisis, case report

## Abstract

Paragangliomas (PGLs) are rare neuroendocrine tumors which overproduce catecholamines (CAs). They are extra-adrenal, catecholamine-secreting tumors occurring outside the adrenal glands. Gastric PGLs originating from extra-adrenal paraganglia are exceptionally rare, and their presentation in geriatric patients further adds to the complexity of diagnosis and management. A 72-year-old male patient presented with enduring left upper abdominal pain and anemia persisting for over a year, and hypertension for six months. Physical examination revealed epigastric discomfort and pallor. Computed tomography scans revealed enlarged lymph nodes in the lesser curvature of the stomach and thickening of the gastric antrum wall with concavity. The patient underwent three cycles of neoadjuvant therapy before radical gastrectomy for gastric cancer. These imaging findings were confirmed during surgery and intraoperative blood pressure was in fluctuation. After the successful resection of the tumor, postoperative pathology confirmed paraganglioma. During postoperative examination, it was observed that the patient’s CAs and their metabolites had returned to within the normal range. Combined with the existing ten literatures, we retrospective report the clinical and pathological characteristics and treatment strategies of the rare gastric paraganglioma.

## 1 Introduction

PGLs are uncommon neuroendocrine tumors originating from neural crest cells within the sympathetic and parasympathetic nervous systems (1). They are typically classified into sympathetic and parasympathetic subtypes, with most sympathetic subtypes being functional, secreting CAs. In patients with catecholamine hypersecretion, most tumors locate at the abdomen and pelvis. Only 3.6% of head and neck PGLs were documented to have catecholamine hypersecretion (2). These tumors represent a subset of pheochromocytomas, comprising approximately 10-18% of all cases, with around 5-10% occurring outside the adrenal glands (3). Gastric PGLs are exceptionally rare, with a five-year survival rate below 5% (4). Histologically, PGLs are characterized by a distinctive “Zellballen” pattern, consisting of spindle-shaped and epithelioid cells. While PGLs have been documented in various anatomical sites such as the head and neck, rectum, liver, and bladder organs, cases of gastric PGLs are exceedingly rare (5–8). Sympathetic PGLs, especially those outside the adrenal glands, are frequently functional and tend to produce norepinephrine rather than epinephrine. Patients often present with hypertension and classical symptoms, including headaches, palpitations, and sweating. Notably, most cases of sympathetic PGLs have a familial component and are linked to genetic mutations (9). Despite the documentation of PGLs in diverse locations, reports of gastric PGLs remain sporadic. Herein, we present a rare case of gastric paraganglioma, contributing to the limited existing literature on this unusual entity.

## 2 Case presentation

**Figure 1:**
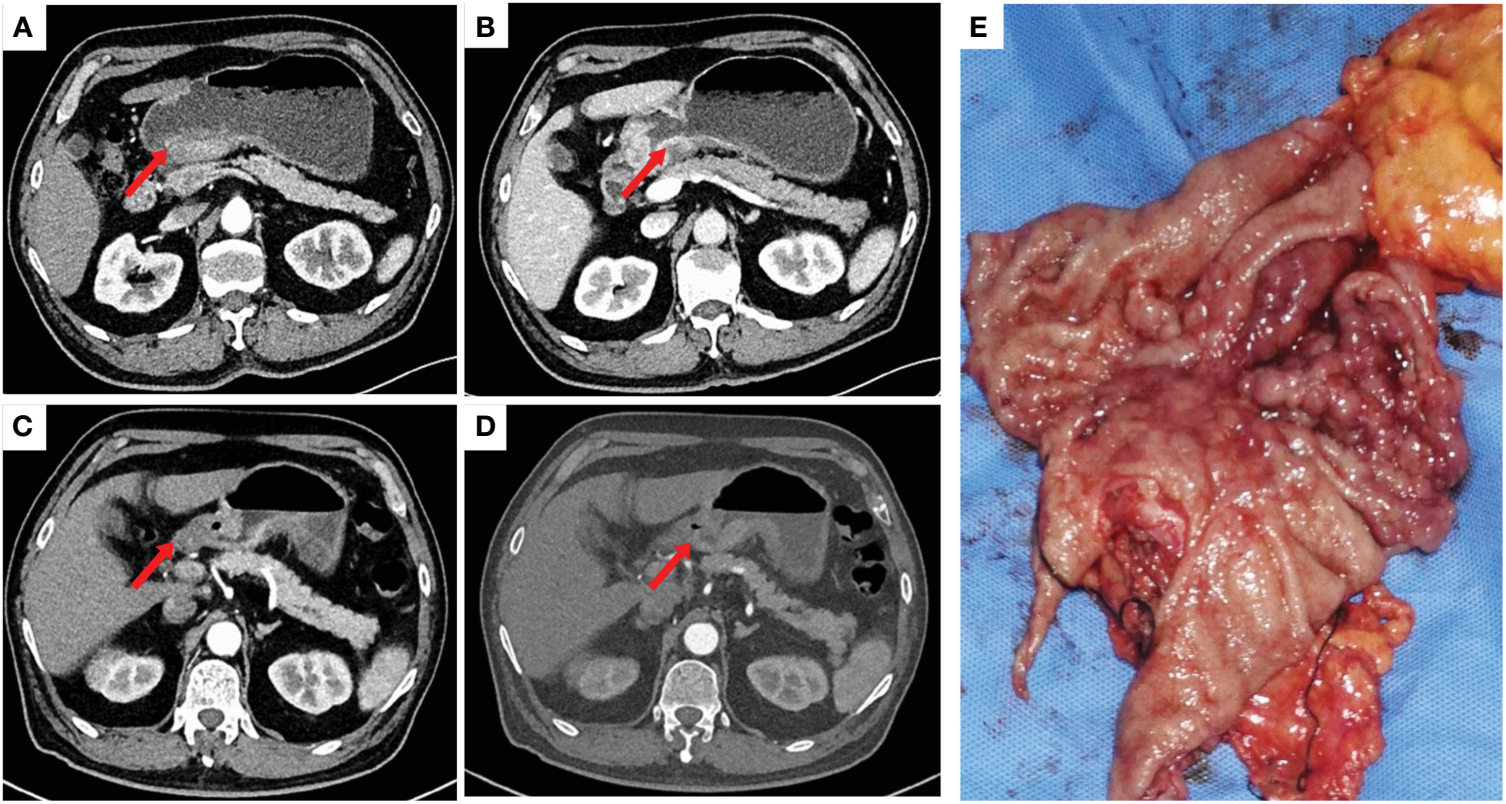
**(A, B)**: Significant thickening of the gastric wall at the antrum was seen before neoadjuvant therapy. **(C, D)**: Significant reduction of visible primary foci and lymph nodes at the gastric antrum after neoadjuvant therapy. **(E)**: Macroscopic presentation of the resected stomach with extensive tumour invasion of the stomach.

**Figure 2:**
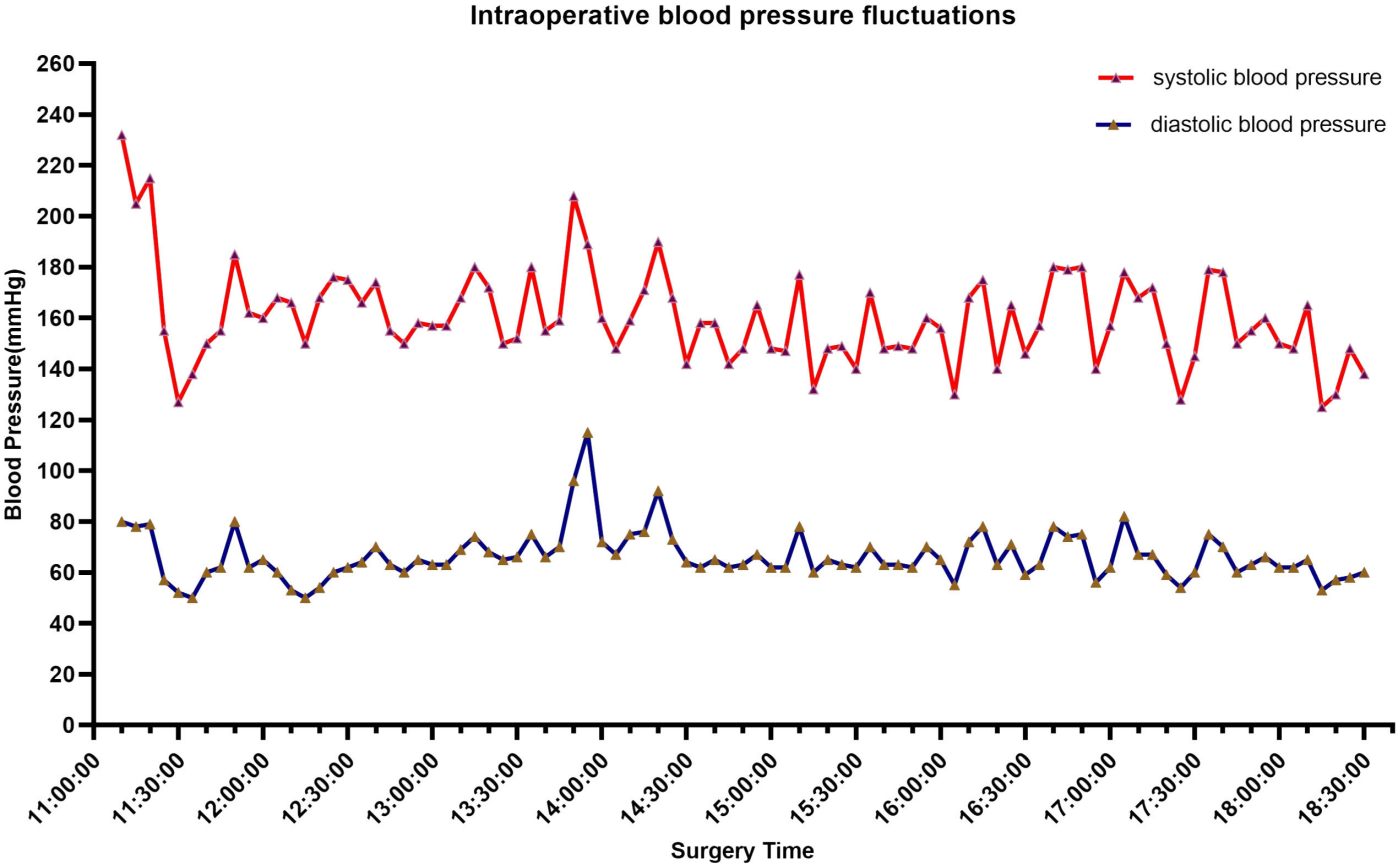
Intraoperative blood pressure fluctuated, with most of the time it was located above 140 mmHg, reaching a maximum of 240 mmHg.

**Figure 3:**
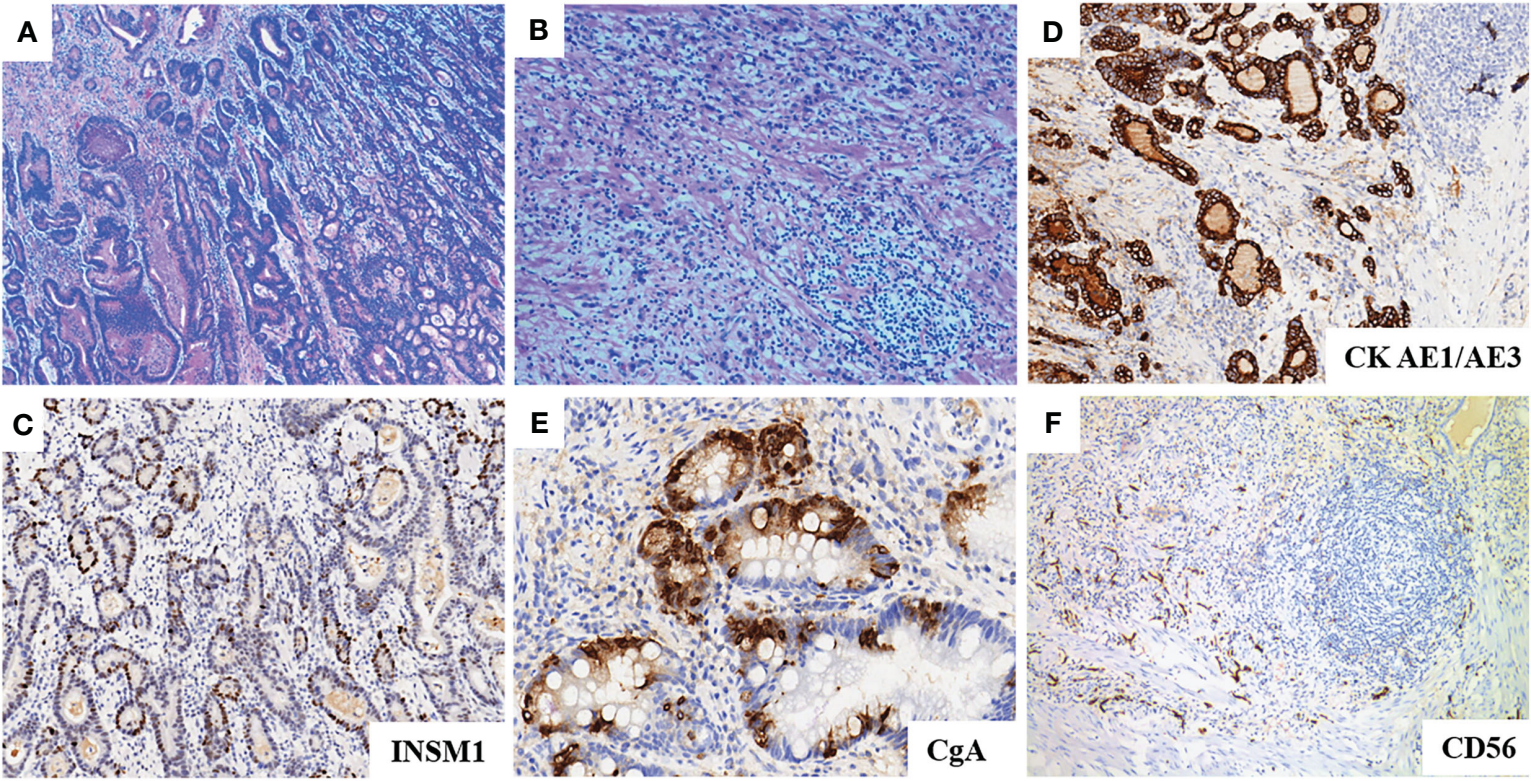
**(A, B)**: HE staining of the tumor showed diffuse growth of paraganglioma cells with moderately to poorly differentiated tubular adenocarcinoma. **(C)**: INSM1 immunohitochemistry shows strong positivity. **(D)**: AE1/AE3 immunohistochemistry shows strong positivity. **(E)**: CgA immunohistochemistry shows focal positivity. **(F)**: CD56 immunohistochemistry shows negativity.

A 72-year-old male patient presented with a persistent history of left upper abdominal pain and anemia spanning over a year. Furthermore, he had developed hypertension six months prior to presentation. Upon physical examination, epigastric discomfort and pallor were evident. The patient underwent gastrointestinal endoscopy with tissue biopsy at other medical center and the pathology suggested adenocarcinoma of the gastric antrum. Therefore, we read the results of the prior diagnostic report and proceeded to perform a subsequent Positron Emission Tomography/Computed Tomography (PET/CT). CT scans revealed enlarged lymph nodes in the lesser curvature of the stomach (**Figures 1A–D**). Subsequent PET/CT imaging unveiled thickening of the gastric antrum wall, displaying a concave configuration, heightened FDG uptake, and invasion of the plasma membrane surface. The tumor was located in the posterior wall, spanning from the antrum to the upper lesser curvature side of the stomach, measuring approximately 10 x 8 cm. The tumor presented with an ulcerated infiltrate and irregularities in the surrounding mucosa, involving the gastric serosa layer (**Figure 1E**). The preliminary diagnosis of gastric cancer was made by the combined examination results. The decision was made to employ a combination of Oxaliplatin and Tegafur chemotherapy as preoperative neoadjuvant therapy. After three cycles of neoadjuvant therapy, the patient demonstrated a reduction in the size of the primary tumor and lymph node shrinkage compared to the pre-treatment period. Then, the patient underwent radical gastrectomy for gastric cancer. However, the patient’s blood pressure continued to fluctuate during the operation, with readings consistently exceeding 140 mmHg, and reaching as high as 240 mmHg (**Figure 2**). The tumor was situated in the posterior wall extending from the gastric antrum to the upper lesser curvature side of the gastric body. During the surgery, the upper margin showed positive results, leading to an extended resection. The upper margin of the upper part of the gastric body still remained positive. Ultimately, a total gastrectomy was performed, with negative margins both above and below the tumor. Simultaneous D2 lymph node dissection was conducted, and the pathological examination revealed 11 lymph nodes on the lesser curvature side of the stomach, with one showing tumor metastasis (1/11). On the greater curvature side, six lymph nodes were observed without tumor metastasis (0/6). No metastasis was detected in the lymph nodes of group 8 (0/2) or group 12 (0/2). Based on these findings, the pTNM staging was determined as pT2N1Mx. Histological examination showed that the nuclei of the tumor cells were round, with obvious nucleoli and clear cytoplasmic demarcation, and the Ki-67 proliferation index was 40%+. CK (AE1/AE3), CgA, and INSM1 staining were positive, while Syn and CD56 staining were negative (**Figures 3 A–F**). These collective findings ultimately led to the diagnosis of gastric paraganglioma. Three days post-surgery, a comprehensive evaluation of blood CAs and their metabolites was conducted. After tumor resection, the levels of norepinephrine (364 pg/ml, normal range 70-1700 pg/ml), epinephrine (22 pg/ml, normal range < 141 pg/ml), methoxy epinep hrine (27.3 pg/ml, normal range < 98 pg/ml), dopamine (14 pg/ml, normal range < 30 pg/ml), vanillylmandelic acid (6.79 ng/ml, normal range < 10.11 ng/ml), and homovanillic acid (10.43 ng/ml, normal range < 33.15 ng/ml) were within the normal range. However, methoxy norepinephrine (181.3 pg/ml, normal range < 164.9 pg/ml) remained persistently elevated. During a follow-up examination, it was observed that the patient’s CAs and their metabolites had returned to within the normal range. The levels of norepinephrine (161 pg/ml), epinephrine (30 pg/ml), methoxy epinephrine (22.6 pg/ml), dopamine (7 pg/ml), vanillic acid (2.76 ng/ml), homovanillic acid (6.7 ng/ml), and methoxy norepinephrine (56.4 pg/ml) were within normal limits. Such a return to baseline levels can be attributed to the effectiveness of the surgical intervention. Finally, the patient was successfully discharged from the hospital. The patient is well and without evidence of metastases 7 months after surgery.

## 3 Discussion

PGLs are now categorized as “metastatic” or “non-metastatic”, shifting away from the traditional benign versus malignant classification (10). Metastatic PGLs exhibit rapid progression and higher mortality rates than their non-metastatic counterparts (3). The evaluation of metastatic risk relies on factors such as tumor size, location, the presence of mutations, dopaminergic phenotype, and the Ki-67 index. Remarkably, clinical presentations of gastric PGLs may present with hypertension, palpitations, and diaphoresis. This highlights the concerning catecholamine hypersecretion frequently observed in PGLs, which can lead to life-threatening consequences. Plasma and urine tests for free CAs and their metabolites are crucial for diagnosing PGLs. Plasma free MNs are more sensitive in diagnosing PGLs compared to measuring CAs, as tumors continuously release metanephrines (MNs) into the bloodstream. Thus, measuring free MNs in plasma or 24-hour urine is the preferred diagnostic test for catecholamine overproduction.

While PGLs have been sporadically reported in various anatomical sites, gastric PGLs have been documented in only ten cases (**Table 1**) (11–19, 21). These included four male and six female patients with a mean age of 64 years. The tumors were predominantly located in the fundus of the stomach in three cases, while others were situated in the anterior gastric wall, posterior gastric wall, and lesser curvature of the stomach. Clinical presentations among the patients primarily included epigastric pain, abdominal mass, vomiting, diarrhea, and various gastrointestinal symptoms. The mean tumor size was approximately 6 cm, with a maximum recorded size of up to 15 cm. Surgical intervention constituted the primary treatment approach, with four patients exhibiting positivity for chromogranin A, neuron-specific enolase, and immunohistochemistry.

**Table 1:** Summary of cases with paragaglioma of stomach.

References	Age	Gender	Symptom	Tumor position	Treatment	IHC result	Size(cm)	Follow-up time (months)
(11)	29	Female	Epigastric pain	Fundus	NA	NA	NA	NA
(12)	55	Male	Epigastric tumor, melaena	Fundus	NA	NA	8	NA
(13)	76	Female	Epigastric distress, melaena	Fundus	surgery	NA	8	48
(14)	49	Male	Haematemesis	Fundus	NA	NA	2.5	NA
(15)	61	Female	Melaena	Lesser curvature	NA	NA	3	NA
(16)	56	Female	Palpable mass	Posterior wall	surgery	GFAP+, NSE+, CgA+, ACTH+, S-100+, CK+	10	48
(17)	85	Male	Abdominal pain	Paragastric region	surgery	Vimentin+, S100+, CgA+, AE1/AE3-,SMA-,CD117-, CD34-,CD99-, HMB-45-, Melan-A-, CD30-	15	22
(18)	82	Female	Abdominal pain	Anterior wall corpuscles	surgery	NSE+, S-100+ GFAP-, AE1/AE3-, CD117-	2	6
(19)	76	Male	No available	Corpuscles	surgery	NSE+, CD117- S100-, CD34e-,SMA-	4	NA
(20)	71	Female	Epigastric pain	Pylorus	surgery	CgA+, S100-, Syn-	6	18

NA, not available.

Genetic testing was performed and analyzed 680 tumor-related genes. We identified three genes associated with paragangliomas (MEN1, EPAS1, MDH2). Meanwhile, missense mutations in EPAS1 and MDH2 which associated with PGLs of the Tricarboxylic acid cycle and hypoxia signaling pathway, result in aberrant regulation and drive the development of paragangliomas (21). The conclusive diagnosis of paraganglioma relies on postoperative pathological examination. The presence of CgA and INSM1 characterizes neuroendocrine tumors, including PGLs. A Ki-67 positivity of 40% indicates higher proliferative activity and increased invasiveness. Differentiating between PGLs and gastrointestinal mesenchymal tumors is difficult due to their co-expression of SMA. However, negative CD117 and positive SMA results can help exclude GISTs as a diagnosis (2). The patient’s pathological examination showed Muc-5AC expression in gastric mucosal cells. Preoperative abdominal CT scan with contrast did not find any adrenal lesions. PET/CT scan results did not indicate the presence of metastatic tumors in the adrenal gland. Mixed gastric adenoendocrine carcinomas are diagnosed based on WHO criteria, with both adenoepithelial and neuroendocrine components comprising at least 30% of the tumor. Studies indicate at least two positive markers (CgA, Syn, CD56) in mixed adenoneuroendocrine carcinomas. In this case, the markedly elevated blood pressure during resection, in conjunction with postoperative immunohistochemistry favored the diagnosis of gastric paraganglioma. Surgical removal of the primary tumor significantly improves patient symptoms and overall survival (2). Meanwhile, Preoperative control of catecholamine levels is essential to reduce the risks associated with intraoperative blood pressure fluctuations.

The clinical presentation of PGLs varies significantly, making early recognition and appropriate treatment crucial to prevent complications and reduce mortality risk.

## Data availability statement

The original contributions presented in the study are included in the article/supplementary material. Further inquiries can be directed to the corresponding author.

## Ethics statement

Written informed consent was obtained from the individual(s) for the publication of any potentially identifiable images or data included in this article.

## Author contributions

CH: Writing – review & editing, Writing – original draft. BL: Writing – review & editing, Writing – original draft. BH: Writing – review & editing, Visualization, Resources. MZ: Data curation, Resources, Writing – review & editing. ZW: Data curation, Writing – review & editing. XZ: Resources, Writing – review & editing, Visualization. FL: Data curation, Writing – review & editing. YH: Data curation, Writing – review & editing. WG: Supervision, Resources, Project administration, Writing – review & editing, Data curation, Conceptualization.
